# VPA Alleviates Neurological Deficits and Restores Gene Expression in a Mouse Model of Rett Syndrome

**DOI:** 10.1371/journal.pone.0100215

**Published:** 2014-06-26

**Authors:** Weixiang Guo, Keita Tsujimura, Maky Otsuka I., Koichiro Irie, Katsuhide Igarashi, Kinichi Nakashima, Xinyu Zhao

**Affiliations:** 1 Department of Neuroscience and Waisman Center, School of Medicine and Public Health, University of Wisconsin, Madison, Wisconsin, United States of America; 2 Department of Stem Cell Biology and Medicine, Graduate School of Medical Sciences, Kyushu University, Fukuoka, Japan; 3 Life Science Tokyo Advanced Research center (L-StaR), Hoshi University School of Pharmacy and Pharmaceutical Science, Tokyo, Japan; University of Insubria, Italy

## Abstract

Rett syndrome (RTT) is a devastating neurodevelopmental disorder that occurs once in every 10,000–15,000 live female births. Despite intensive research, no effective cure is yet available. Valproic acid (VPA) has been used widely to treat mood disorder, epilepsy, and a growing number of other disorders. In limited clinical studies, VPA has also been used to control seizure in RTT patients with promising albeit somewhat unclear efficacy. In this study we tested the effect of VPA on the neurological symptoms of RTT and discovered that short-term VPA treatment during the symptomatic period could reduce neurological symptoms in RTT mice. We found that VPA restores the expression of a subset of genes in RTT mouse brains, and these genes clustered in neurological disease and developmental disorder networks. Our data suggest that VPA could be used as a drug to alleviate RTT symptoms.

## Introduction

Rett syndrome (RTT) is a devastating neurodevelopmental disorder that occurs once in every 10,000–15,000 live female births. RTT patients develop normally until 6 to 18 months of age, but then regress rapidly, experiencing a wide range of neurological symptoms, including seizures, ataxia, and stereotypical hand movements with impairment of communication and cognition [1]. Seizure activity is common and reportedly occurs in up to 80% of patients.

RTT results largely from functional mutations in the X-linked *MECP2* gene [2], which encodes a methylated CpG-binding protein that regulates transcription via epigenetic mechanisms [3]. Mutations and duplications of MeCP2 are also found in several other developmental disorders, including autism, demonstrating the functional importance of MeCP2 [4], [5]. *Mecp2* null mutant (KO) mice develop similar symptoms as those seen in RTT patients; these mice have been used widely to study the etiology of human RTT [6], [7], [8]. Using RTT mice, we and others have shown that MeCP2 deficiency leads to altered expression of downstream effectors, resulting in impaired neuronal differentiation and maturation [9], [10], [11]. During the past decade, there have been extensive efforts devoted to understanding and treating RTT. However, for the most part we still lack effective and safe treatments.

Valproic acid (VPA) has been used clinically for decades as a treatment for mood disorders and seizures [12], [13], [14]. It was later also found to be an inhibitor for histone deacetylases, which are known to repress the expression of many genes [15], [16]. Therefore, VPA could potentially affect a large number of genes, although its impact might be specific to different cell types. Despite the fact that its mechanism of action is not fully clear, VPA has been used or considered as a drug for a number of neurological diseases, including spinal muscular atrophy (SMA), Parkinson's disease, Huntington's disease, migraine, and dementia, as well as other diseases such as cancer and HIV infection. Since VPA exhibits broad efficacy but only mild and transient side effects, to date it has been used in more than 200 clinical trials for various diseases [12].

VPA has also been used to treat RTT patients, but mostly for seizure control [17]. Although one study found no beneficial effects [18], another study demonstrated a significant reduction in seizures in RTT patients by VPA [19]. Some limited data have also revealed that VPA can improve behavioral deficits other than seizure in RTT, including verbal fluency [20] and decreased risk of fracture [21]. However, whether VPA can improve neurological symptoms has not been systematically assessed. VPA is known to restore MeCP2 deficiency-induced protein changes in a cultured cell system [22], yet whether VPA treatment can restore gene expression in MeCP2-deficient brains has not been tested.

In this study, we aimed to evaluate the therapeutic effect of VPA on symptomatic RTT mice. We treated Mecp2 KO mice with VPA at a peak of neurological symptoms and found that VPA could alleviate RTT-associated neurological symptoms. In addition, VPA partially restores global gene expression changes in MeCP2 KO mice. Interestingly, VPA specifically affects genes in the pathway related to neurological diseases. Thus our data support a potential therapeutic role for VPA in the treatment of RTT.

## Materials and Methods

### Ethics Statement

All animal procedures were performed according to protocols approved by the University of New Mexico and University of Wisconsin Animal Care and Use Committee. The *Mecp2* KO mice (*Mecp2^tm1.1Jae^*) used in this study were created by deleting exon 3 containing the MBD domain of Mecp2 [23]. These mice have been bred over 40 generations on the ICR background. They start to show neurological symptoms between 5 and 7 weeks of age, and many die before 10 weeks of age, although some live as long as 17 weeks of age.

### VPA Treatment

**Figure 1:**
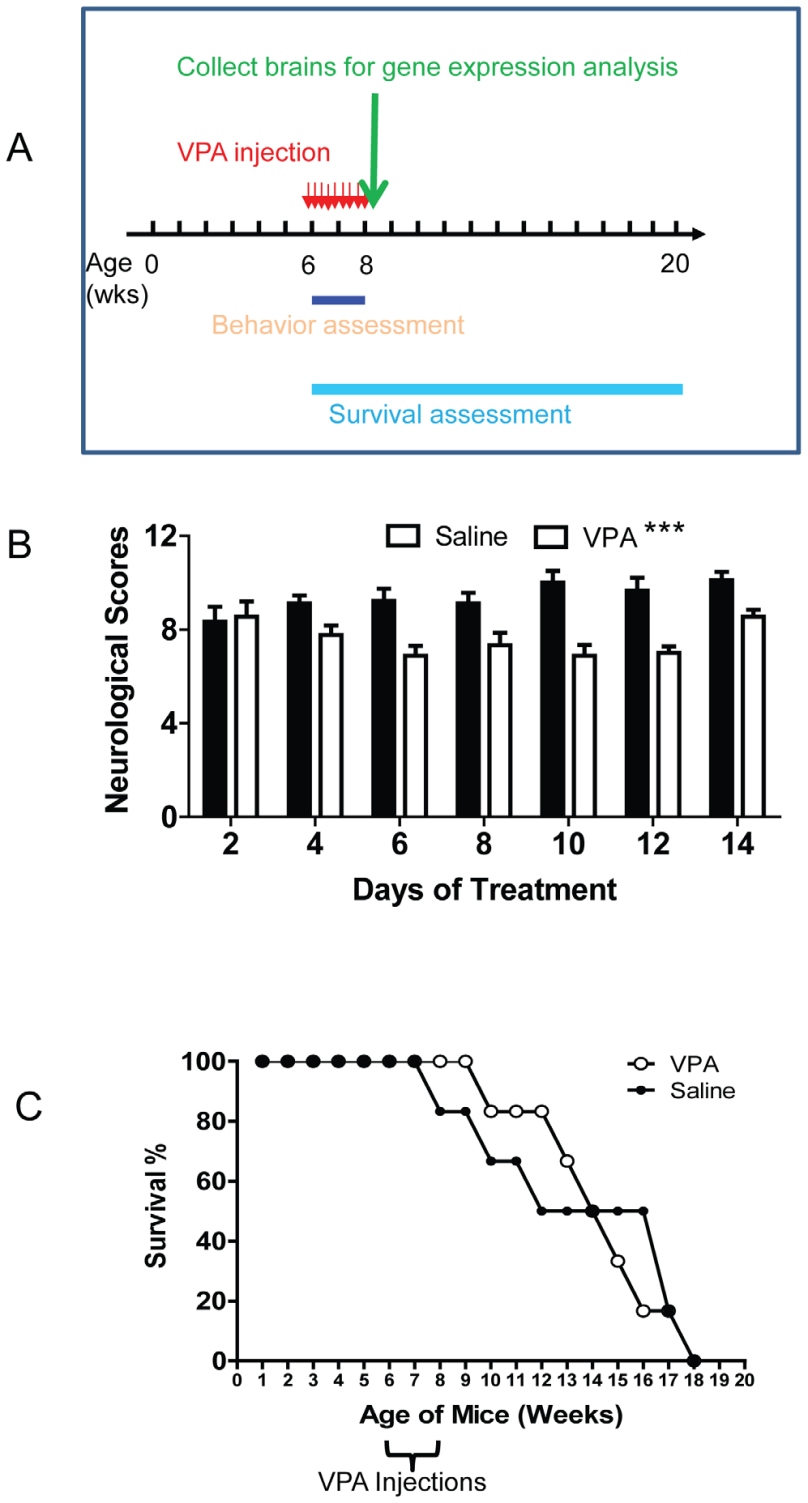
VPA treatment rescues certain neurological symptoms in MeCP2 KO mice. (A) Schematic drawing shows the timeline of experiments. (B) Neurological symptom scores of saline (control) and VPA-treated MeCP2 KO mice (n = 9 per group, two-way ANOVA with repeated measure, time and treatment interaction: F_6,96_ = 3.079, p = 0.0084; VPA treatment: F_1,96_ = 28.22, p<0.0001). (C) VPA treatment has small but noticeable effects on the lifespan of MeCP2 KO mice. ***, p<0.001

When mice reached 6 weeks old, they received daily injections of VPA (300 mg/kg; make 50 mg/ml VPA in saline) for 2 weeks (Fig 1). Three batches of mice were used. The first batch of mice included 3 groups of mice: WT control (n = 3), KO treated with VPA (KO+VPA, n = 5), and KO treated with saline (KO+saline, n = 4). We first did behavioral assessments during the VPA injection period. Immediately after the last injection, we dissected the brains from 3 mice per group and froze them in liquid N_2_ for RNA isolation. The rest of the mice were used for survival analysis. The second and third batches of mice included KO+VPA (n = 4) and KO+saline (n = 5); we did behavioral assessments during the injection period, and then we recorded the survival of mice. Therefore, total 9 KO+VPA and 9 KO+saline mice were assessed for behavioral symptom (Figure 1B) and 6 KO+VPA and 6 KO+saline mice were assessed for survival (Figure 1C).

### Neurological Symptom Assessment

This assessment was carried out based on a published method [24]. We evaluated 6 core symptoms of RTT and scored the severity of symptoms: Score 0 indicated an absence of symptoms (wild-type all had 0); Score 1 indicated the presence of symptoms; Score 2 designated severe symptoms. Mice were also weighed at each scoring session. The sum of scores in all categories was used to represent the severity of symptoms. The symptoms we assessed and scoring criteria are as follow:

Mobility: The mouse is observed when placed on a bench, then when handled gently. Score 0 = same as wild-type. Score 1 = reduced movement when compared to wild-type: extended freezing period when first placed on bench and longer periods spent immobile. Score 2 = no spontaneous movement when placed on the bench; mouse can move in response to a gentle prod or a food pellet placed nearby. (Note: mice may become more active when in their own cage environment.)

Gait: Score 0 = same as wild-type. Score 1 = hind legs are spread wider than wild-type when walking or running with reduced pelvic elevation, resulting in a “waddling” gait. Score 2 = more severe abnormalities: tremor when feet are lifted, walks backwards or “bunny hops” by lifting both rear feet at once.

Hind limb clasping: Mouse observed when suspended by holding base of the tail. Score 0 = legs splayed outwards. Score 1 = hind limbs are drawn towards each other (without touching) or one leg is drawn in to the body. Score 2 = both legs are pulled in tightly, either touching each other or touching the body.

Tremor: Mouse observed while standing on the flat palm of the hand. Score 0 = no tremor. Score 1 = intermittent mild tremor. Score 2 = continuous tremor or intermittent violent tremor.

Breathing: Movement of flanks observed while animal is standing still. Score 0 = normal breathing. Score 1 = periods of regular breathing interspersed with short periods of more rapid breathing or with pauses in breathing. Score 2* = very irregular breathing: gasping or panting.

General condition: Mouse observed for indicators of general wellbeing, such as coat condition, eyes, body stance. Score 0 = clean shiny coat, clear eyes, normal stance. Score 1 = eyes dull, coat dull/un-groomed, somewhat hunched stance. Score 2* = eyes crusted or narrowed, piloerection, hunched posture.

### Gene Expression Microarray Analysis

Half of the brain tissues from the first batch of mice (n = 3 mice/condition) were used for microarray analysis. Microarray analysis was performed following the manufacturer's instructions. First, total RNAs were purified using an RNeasy Mini kit (Qiagen) from half brains. First strand cDNAs were synthesized by incubating 5 µg of total RNA with SuperScript II reverse transcriptase (Invitrogen). After second-strand synthesis, the double-stranded cDNAs were purified using a MinElute Reaction Cleanup Kit (Qiagen) and labeled by in vitro transcription using a BioArray High Yield RNA transcript labeling kit (Enzo Life Sciences, Farmingdale, NY, USA). The labeled cRNA was then purified using an RNeasy Mini kit (Qiagen) and treated with fragmentation buffer at 94°C for 35 min. For hybridization to a GeneChip Mouse Genome 430 2.0 Array (Affymetrix), 7.5 µg of fragmented cRNA probe was incubated with 50 pM control oligonucleotide B2, 1x eukaryotic hybridization control (1.5 pM BioB, five pM BioC, 25 pM BioD and 100 pM Cre), 0.1 mg/mL herring sperm DNA, 0.5 mg/mL acetylated BSA, and 1X manufacturer-recommended hybridization buffer in a 45°C rotator for 16 h. Washing and staining were performed in a GeneChip Fluidics Station (Affymetrix). The phycoerythrin-stained arrays were scanned as digital image files that were then analyzed with GeneChip Operating Software (Affymetrix). We selected the probe sets with the fold change of MeCP2KO-saline to wild-type more than 1.2 and a p-value under 0.05. Pathway analysis was performed using the Ingenuity Pathways Analysis software (Ingenuity Systems). Gene Ontology (GO) analyses were performed using Genespring software (Agilent Technologies)

### Real-time PCR

Total RNAs from half brains (n = 3/condition) were prepared by using RNA purification kits (Qiagen). Real-time RT-PCR TaqMan probes and reaction reagents were purchased from Applied Biosystems. Reactions were performed according to manuals from the manufacturer by using StepOnePlus Real-time PCR system (Applied Biosystems). All results were normalized to levels of the GAPDH gene. Catalog numbers for the probes are: GAPDH (Mm03302249_g1), zinc finger with KRAB and SCAN domains 1 (Zkscan1) (Mm00551752_m1), AE binding protein 2 (Aebp2) (Mm01267857_m1), contactin 1 (Cntn1) (Mm00514374_m1), and potassium inwardly-rectifying channel, subfamily J, member 16 (Kcnj16) (Mm04208325_m1).

### Data deposition

The datasets that were generated and reported in this paper have been deposited into the National Center for Biotechnology Information GEO database through accession number GSE56780.

## Results

### VPA treatment can alleviate neurological symptoms in MeCP2-deficient mice

Since Mecp2 mutant mice on C57B/L6 background are difficult to breed, we bred the Mecp2 mutant (Jaenisch/MIT) line [23] onto ICR background for over 40 generations. The Mecp2 heterozygote ICR female mice gave birth to significantly more surviving pups compared to their C57B/L6 counterparts therefore we used ICR mice for this study. Mecp2 KO male mice on the ICR genetic background started showing symptoms at about 5 weeks of age, and many of them died between 8 and 10 weeks of age, which is similar to Mecp2 KO mice on C57B/L6 background. However. Some of the Mecp2 KO ICR males lived up to 17 weeks, which is significantly longer than their C57B/6 counterparts. To determine whether VPA treatment might have a therapeutic effect on symptomatic KO mice, we decided to treat mice between 6 to 8 weeks of age, when all of them were showing clear RTT symptom, but before they were too sick to receive Intraperitoneal (i.p.) injections (Fig. 1A). Before daily injection of VPA, we first assessed the severity of neurological symptoms that are characteristics of RTT, including mobility, gait, hind limb clasping, tremor, breathing, and their general condition. We applied a published scoring system by setting the severity scale from 0 to 2: 0 is absent, 1 is present, and 2 is severe for each symptom [24]. Based on these criteria, all wild-type (WT) mice showed no symptoms, and therefore scored as 0. We found that the severity of neurological symptoms in saline-injected KO mice did not change significantly during the 2-week assessment and injection period (Figure 1B, black bar, one-way ANOVA with repeated measure, F_6,48_ = 2.085, p = 0.0724). On the other hand, the neurological scores of VPA-treated KO mice exhibited significant changes during the 2-week period (Figure 1B, white bar, one-way ANOVA with repeated measure, F_6,48_ = 2.685, p = 0.025), suggesting effects from VPA treatment on symptom development. More importantly, VPA treatment led to a significant reduction in the neurological severity scores compared to saline-treated KO mice (Fig. 1B, two-way ANOVA with repeated measure, time and treatment interaction: F_6,96_ = 3.079, p = 0.0084; VPA treatment: F_1,96_ = 28.22, p<0.0001). Among all six symptoms analyzed, gait (F_1,96_ = 8.544, p = 0.01), tremor (F_1,96_ = 16.42, p = 0.0009), breathing (F_1,96_ = 14.22, p = 0.0017), and general health condition (F_1,96_ = 5.511, p = 0.0321) showed significant improvement in VPA treated KO mice (Figure S1 in File S1). Mobility showed strong time and treatment interaction (F_6,96_ = 5.657, p<0.0001) without significant effect by treatment alone. There was no significant difference in body weight between the treatment groups. We also assessed the survival of these mice both during and after the 2-week VPA treatment period. We found that although VPA treatment delayed the death of some of these KO mice, it had no significant effect on the overall lifespan of KO mice (Fig. 1C). Therefore, a short 2-week VPA treatment during the peak symptomatic period can reduce several key RTT neurological symptoms in a RTT mouse model.

### VPA can rescue gene expression changes

To determine the molecular basis of symptom improvement, we collected brains from Mecp2 KO mice treated with VPA (KO+VPA) or saline (KO+Saline) and WT mice at one day after the last VPA injection. We isolated RNA from these brains and subjected them to microarray analysis. The biological triplicates within the same experimental groups showed excellent reproducibility (Figure S2 in File S1).

**Figure 2:**
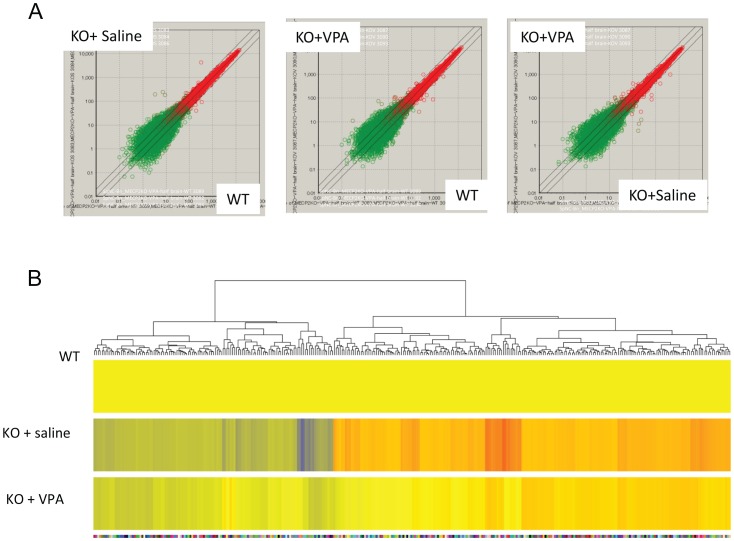
VPA treatment restores the expression of a subset of genes in MeCP2 KO brains. (A) Scatter plots showing differential gene expression profiles among experimental conditions. (B) Heatmap showing 33 genes with >1.2-fold changes in the MeCP2 KO compared to WT brains and that were restored to <1.2-fold changes upon VPA treatment.

Comparison among different experimental groups revealed significant expression changes in subsets of genes resulting from MeCP2 deficiency (KO+saline vs. WT), as well as restoration by VPA (KO+VPA vs. WT) (Table S1). To evaluate whether VPA could restore gene expression caused by MeCP2 deficiency, we selected genes that are significantly altered in MeCP2 KO mice (KO+saline vs WT:>1.2-fold, p<0.05), but are restored by VPA treatment (KO+VPA vs. WT:<1.2-fold). The heat map demonstrates that 310 genes presented by 333 probe sets met these criteria (Fig 2B). Expression of some of these genes, such as *Zkscan1*, was reduced to near WT levels by VPA. Therefore, VPA can normalize the expression of a subset of genes to nearly WT levels in MeCP2-deficient brains.

### VPA treatment restores expression of a subset of genes involved in neurological disorders

**Figure 3:**
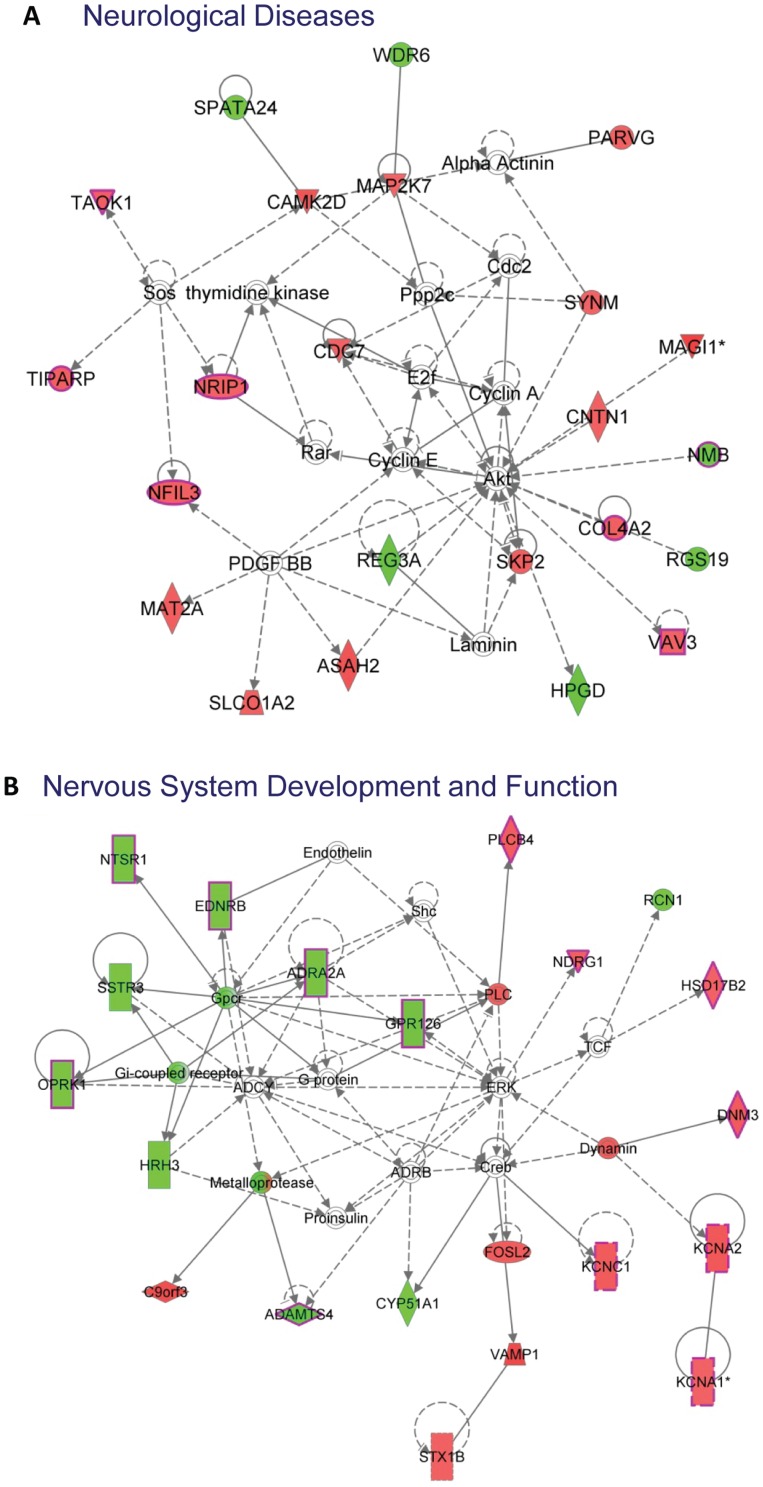
Ingenuity Pathway Analysis showing the two top networks restored by VPA in MeCP2 KO brains. The 310 gene restrored by VPA in MeCP2 KO brains are mostly categorized into two networks: Neurological Disease network (A) and Nervous System Development and Function network (B). The colors indicate the direction of gene changes. Red, KO is higher than WT. Green, KO is lower than WT. The shapes of the boxes represent the functional categories of the genes. Inverted triangle: Kinase; Horizontally oval: Transcription Regulator, Vertical diamond: Enzyme, Square: Cytokine; Circle: Other; Bordered circle: Complex/Group, Vertically rectangle: G-protein Coupled Receptor; Horizontally diamond: Peptidase, Triangle: Phospatase.

To determine the types of genes whose expression was restored by VPA in the context of MeCP2 deficiency, we subjected these 310 genes to Ingenuity Pathway Analysis and found that the top networks affected by VPA (Table 1 and Table S2) include Neurological Diseases (Fig 3A) and Nervous System Development and Function (Fig 3B). When we analyzed the biological functions of these 310 genes, they were categorized into three main biological function groups: “Diseases and Disorders,” “Molecular and Cellular Functions,” and “Physiological System Development and Function” (Table 2). Interestingly, the “Diseases and Disorders” group contains the largest number of genes, with 83 genes in the category of “Neurological Disease” (Table 3). A Gene Ontology (GO) analysis also showed many of these genes are involved in important cellular functions (Table S3). Thus VPA restored genes in MeCP2-deficient brains mainly in the category of neurological disease and brain development.

**Table 1:** Top Networks Restored by VPA Treatment.

ID	Top Diseases and Functions	Score
1	Cancer, Neurological Disease, Connective Tissue Disorders	40
2	Organismal Injury and Abnormalities, Cellular Growth and Proliferation, Cellular Movement	35
3	Nervous System Development and Function, Molecular Transport, Behavior	35
4	Cellular Function and Maintenance, Hematological System Development and Function, Inflammatory Response	32
5	Cellular Assembly and Organization, Cellular Function and Maintenance, Cell Morphology	30

**Table 2:** Top Disease and Bio Function Groups Restored by VPA.

Diseases and Disorders
Name	p-value #	Molecules
Neurological Disease	3.34E-08-1.74E-02	83
Cancer	5.18E-07-1.71E-02	150
Cardiovascular Disease	2.98E-06-1.71E-02	44
Organismal Injury and Abnormalities	2.69E-05-1.71E-02	42
Psychological Disorders	3.12E-05-1.33E-02	43

Molecular and Cellular Functions
Name	p-value #	Molecules
Cell Morphology	9.35E-06-1.82E-02	69
Molecular Transport	1.05E-04-1.59E-02	60
Lipid Metabolism	1.45E-04-1.57E-02	29
Small Molecule Biochemistry	1.45E-04-1.57E-02	45
Cellular Assembly and Organization	1.79E-04-1.61E-02	58

Physiological System Development and Function
Name	p-value #	Molecules
Behavior	1.43E-07-1.34E-02	45
Cardiovascular System Development and Function	2.33E-04-1.81E-02	43
Organismal Survival	2.34E-04-5.76E-03	65
Organismal Development	3.66E-04-1.82E-02	60
Nervous System Development and Function	4.78E-04-1.82E-02	62

**Table 3:** Neurological Disease Genes Restored by VPA.

Gene Symbol	Gene Title
2010111I01Rik	RIKEN cDNA 2010111I01 gene
Adamts4	a disintegrin-like and metallopeptidase (reprolysin type) with thrombospondin type 1 motif, 4
Adamtsl1	ADAMTS-like 1
Adra2a	adrenergic receptor, alpha 2a
Bcl11a	B cell CLL/lymphoma 11A (zinc finger protein)
Bub3	budding uninhibited by benzimidazoles 3 homolog (S. cerevisiae)
Cdc14b	CDC14 cell division cycle 14B
Cds1	CDP-diacylglycerol synthase 1
Chrna6	cholinergic receptor, nicotinic, alpha polypeptide 6
Cntn1	contactin 1
Col3a1	collagen, type III, alpha 1
Col4a2	collagen, type IV, alpha 2
Col5a2	collagen, type V, alpha 2
Crym	crystallin, mu
Cyp51	cytochrome P450, family 51
Dab1	disabled 1
Dnajc1	DnaJ (Hsp40) homolog, subfamily C, member 1
Dusp5	dual specificity phosphatase 5
Ednrb	endothelin receptor type B
Efna5	ephrin A5
Fat4	FAT tumor suppressor homolog 4 (Drosophila)
Fbln1	fibulin 1
Fhdc1	FH2 domain containing 1
Fos	FBJ osteosarcoma oncogene
Foxp1	forkhead box P1
Gabra3	gamma-aminobutyric acid (GABA) A receptor, subunit alpha 3
Galnt7	UDP-N-acetyl-alpha-D-galactosamine: polypeptide N-acetylgalactosaminyltransferase 7
Glra1	glycine receptor, alpha 1 subunit
Gpr126	G protein-coupled receptor 126
Hcn1	hyperpolarization-activated, cyclic nucleotide-gated K+1
Hrh3	histamine receptor H3
Ier5	immediate early response 5
Kazn	kazrin, periplakin interacting protein
Kcna1	potassium voltage-gated channel, shaker-related subfamily, member 1
Kcna2	potassium voltage-gated channel, shaker-related subfamily, member 2
Kcnc1	potassium voltage gated channel, Shaw-related subfamily, member 1
Krit1	KRIT1, ankyrin repeat containing
Ldlr	low density lipoprotein receptor
Lgals1	lectin, galactose binding, soluble 1

Gene Symbol	Gene Title
Malat1	metastasis associated lung adenocarcinoma transcript 1 (non-coding RNA)
Marcksl1	MARCKS-like 1
Met	met proto-oncogene
Mtmr9	myotubularin related protein 9
Ndrg1	N-myc downstream regulated gene 1
Nfatc2	nuclear factor of activated T cells, cytoplasmic, calcineurin dependent 2
Nfil3	nuclear factor, interleukin 3, regulated
Nmb	neuromedin B
Nppa	natriuretic peptide type A
Nrip1	nuclear receptor interacting protein 1
Ntrk3	neurotrophic tyrosine kinase, receptor, type 3
Oprk1	opioid receptor, kappa 1
Paqr6	progestin and adipoQ receptor family member VI
Pclo	piccolo (presynaptic cytomatrix protein)
Pcsk1	proprotein convertase subtilisin/kexin type 1
Pim3	proviral integration site 3
Plcb4	phospholipase C, beta 4
Pnkd	paroxysmal nonkinesiogenic dyskinesia
Ppargc1a	peroxisome proliferative activated receptor, gamma, coactivator 1 alpha
Ppp1r1a	protein phosphatase 1, regulatory (inhibitor) subunit 1A
Prdm2	PR domain containing 2, with ZNF domain
Prkcg	protein kinase C, gamma
Rims3	regulating synaptic membrane exocytosis 3
Scn1a	sodium channel, voltage-gated, type I, alpha
Sema3f	sema domain, immunoglobulin domain (Ig), short basic domain, secreted, (semaphorin) 3F
Skp2	S-phase kinase-associated protein 2 (p45)
Slc16a2	solute carrier family 16 (monocarboxylic acid transporters), member 2
Slco3a1	solute carrier organic anion transporter family, member 3a1
Smpx	small muscle protein, X-linked
Sst	somatostatin
Sstr3	somatostatin receptor 3
Sv2c	synaptic vesicle glycoprotein 2c
Taok1	TAO kinase 1
Thbs3	thrombospondin 3
Tiparp	TCDD-inducible poly(ADP-ribose) polymerase
Tnc	tenascin C
Tnnt1	troponin T1, skeletal, slow
Trps1	trichorhinophalangeal syndrome I (human)
Tubb2b	tubulin, beta 2B class IIB
Vamp1	vesicle-associated membrane protein 1
Vav3	vav 3 oncogene
Vcam1	vascular cell adhesion molecule 1

**Figure 4:**
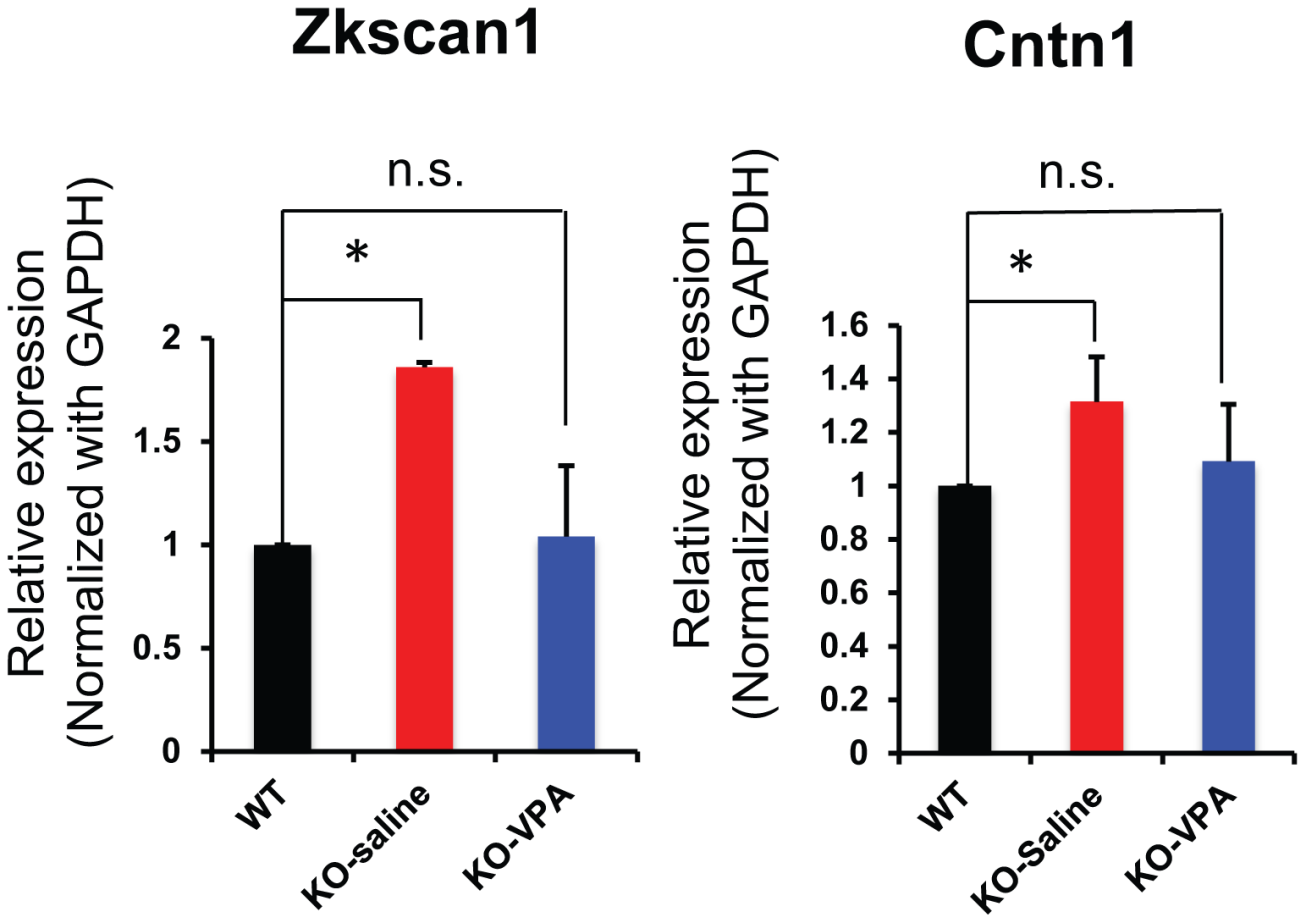
Quantitative PCR data showing restoration to WT levels of Zkscan1 and Cntn1 in MeCP2 KO brains by VPA treatment. The levels of Zkscan1 and Cntn1 mRNA were reduced to the WT levels by VPA treatment. *, p<0.05 (n = 3).

We next used quantitative PCR to assess the expression levels of some of these VPA-restored genes (Figure 2B and Table S1). We confirmed that VPA treatment indeed restored the expression levels of Zkscan1, a transcription factor important for cell differentiation, and Cntn1, a cell adhesion protein with a central role in neuronal growth cones and axon guidance (Fig 4). We then selected a few interesting genes that also showed VPA restoration, but were just below the cut-off of our stringent data analysis criteria. Among these candidates that also showed restoration by VPA were Aebp2, a DNA-binding epigenetic regulator, and Kcnj16, a potassium channel (Figure S3 in File S1). Therefore, in MeCP2-defiicent brains, VPA could restore the expression of a subset of genes involved in neurological disease and brain development.

## Discussion

In this study we explored whether VPA treatment could alleviate neurological symptoms of RTT. We discovered that VPA treatment during the symptomatic stage of disease significantly reduced neurological deficits in RTT mice. We also showed that VPA can restore the expression of a subset of neurological disease-related genes resulting from MeCP2 deficiency.

Although VPA has been used to treat seizures in a small number of RTT patients, its effect on neurological symptoms has not been well documented. The fact that treatment with VPA during the peak symptomatic stage of the disease could significantly improve neurological symptoms in MeCP2 KO mice is exciting. Our data suggest VPA may be a promising drug for the treatment of RTT. One remaining question is whether VPA treatment either before or during the early onset of symptoms may lead to additional improvements. Moreover, we only treated mice for two weeks. It is possible that longer treatment might bring more relief of symptoms and a longer lifespan. However, i.p. injection at the symptomatic stage may cause more stress to the animals, so the VPA injection route will have to be optimized (e.g. oral administration).

Since VPA inhibits HDACs, which are known to be involved in the suppression of many genes, we expected to detect a great many genes whose expression level is altered by VPA in the brain; however, we could only find 310 genes with a significant restoration of their expression levels in response to the VPA treatment. This could be due in part to the fact that we used brain tissues containing different cell types, which may respond to VPA differently. It is also likely that the brain's compensatory changes may mask the effect of VPA. Nevertheless, it is worth noting that the 310 genes restored by VPA are clustered in the network of Neurological Disease and Nervous System Development (Figure 3, Table 1, Table S2), and 83 of these genes are in the Disease and Disorder functional group (Table 2). Many of these genes have been linked to human diseases. For example, a mutation in CNTN1, a neural adhesion protein, leads to a familial form of lethal congenital myopathy [25]. Transcription factor FOXP1 deletion and overexpression are both linked to autism [26], [27], and transcription factor ZKSCAN1 is associated with Wolf-Hirschhorn syndrome with intellectual disability (www.genecards.org). The molecular and cellular biological function group restored by VPA included molecular transport, lipid metabolism, small molecule biochemistry, and cell morphology. The restoration of cell morphology may be an important application for rescuing the impaired dendritic development in MeCP2-deficient brains that we and others have observed [9]. The potential action of VPA in molecular transport, metabolism, and biochemistry is also very interesting because MeCP2 was recently found to play a role in RNA splicing [28], [29] and nuclear size determination [30]. In addition, as a next step, it would be interesting to explore the cell type-specific changes of some of the candidate genes we have discovered. We also believe we may have missed some other genes restored by VPA due to the stringency of our data analysis. For example, Aebp2, an epigenetic DNA-binding protein involved in Hirschsprung's disease and Waardenburg syndrome [31], and Kcnj16, a potassium channel involved in respiratory response to hypoxia during breathing [32], showed VPA-restored expression changes, but the p value was greater than 0.05. It is likely that changes in other genes were masked by the complexity of brain tissues we used. Previous studies have found hundreds of genes are changed in the hypothalamus or cerebellum of MeCP2 KO mice [33], [34]. A recent study has identified 127 genes were altered in the striatum of MeCP2 KO mice [35]. A comparison of our data and these data showed a small percentage of the genes are shared. For example, among 383 genes altered in the hypothalamus of Mecp KO mice[34], 8 of them are in the 621 genes we found altered in the whole brain of Mecp2 KO mice and 5 of them are found in the 310 genes restored by VPA. It is not surprising that only small percentage of genes overlap among these data sets given the different original of the tissues and mixed composition of cells in these brain regions. Future studies using pure populations of cells will likely reduce false negatives and uncover more VPA-restored genes in specific brain cell types. VPA may affect different sets of genes in specific cell types, which will be a valuable avenue of study to pursue in the future.

## Supporting Information

Table S1 **VPA restored Genes in MeCP2 KO Brains.**
(XLS)

Table S2 **Top Networks and Genes Restored by VPA Treatment.**
(XLS)

Table S3 **Gene Ontology Analysis of VPA-Restored Genes in MeCP2 KO Brains.**
(XLSX)

File S1 **This file contains 3 supplemental figures (Figure S1, Figure S2, and Figure S3). Figure S1.** VPA treatment rescues certain pathological symptoms in MeCP2 KO mice. **Figure S2**. Scatter plots showing reproducibility in gene expression profiles among biological triplicates within each experimental condition. **Figure S3**. Quantitative PCR data showing restoration of Aebp2 and Kcnj16 genes in MeCP2 KO brains by VPA treatment.
(PDF)
