# Supplementary material for: VPA Alleviates Neurological Deficits and Restores Gene Expression in a Mouse Model of Rett Syndrome
Source: PLoS One. 2014 Jun 26;9(6):e100215. doi: 10.1371/journal.pone.0100215 (PMC4072629; doi:10.1371/journal.pone.0100215)
Supplement: File S1 — This file contains 3 supplemental figures (Figure S1, Figure S2, and Figure S3). Figure S1. VPA treatment rescues certain pathological symptoms in MeCP2 KO mice. Figure S2. Scatter plots showing reproducibility in gene expression profiles among biological triplicates within each experimental condition. Figure S3. Quantitative PCR data showing restoration of Aebp2 and Kcnj16 genes in MeCP2 KO brains by VPA treatment. (PDF) [file pone.0100215.s004.pdf]

Figure S1:

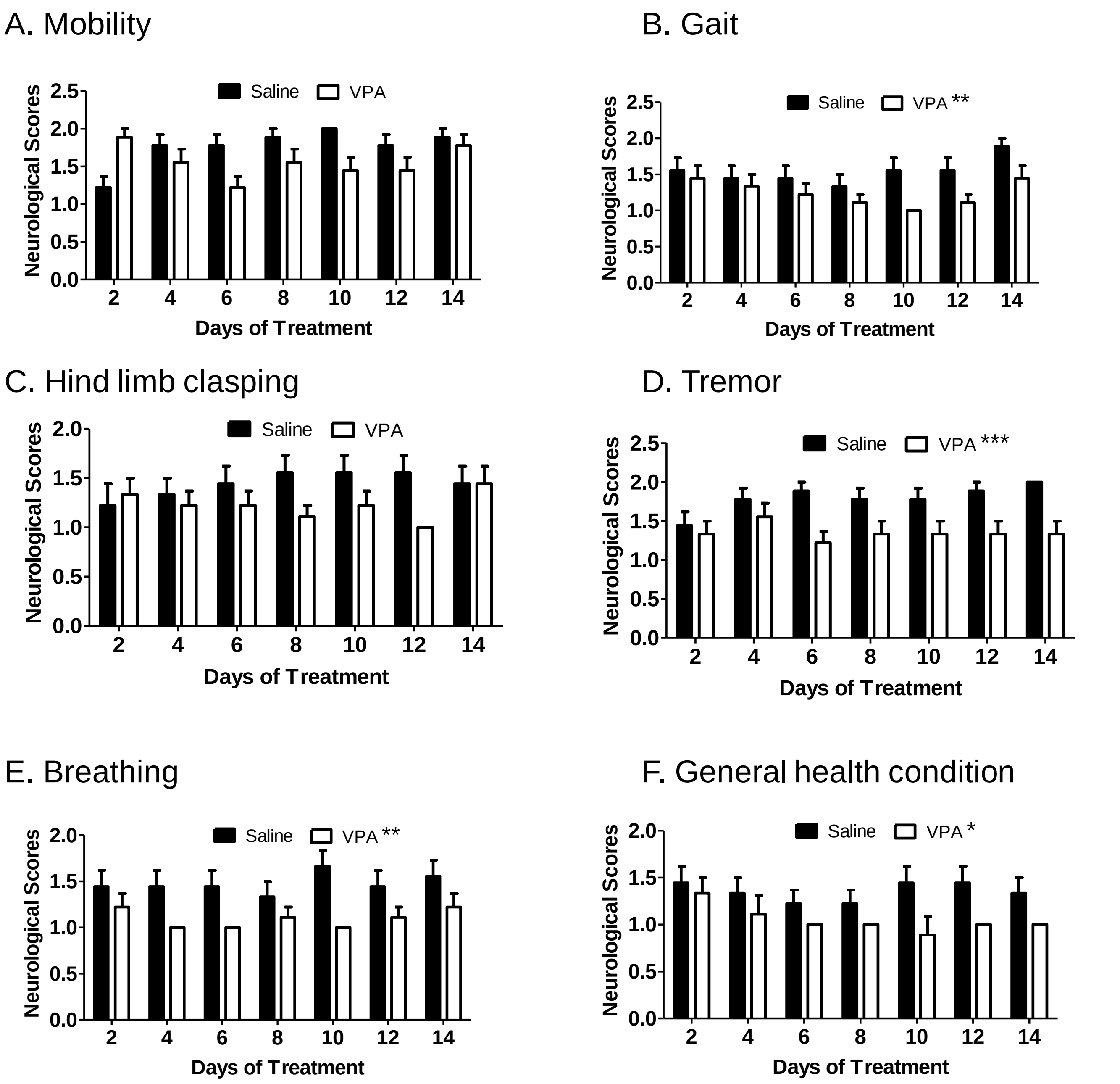

Figure S1. . VPA treatment rescues certain pathological symptoms in MeCP2 KO mice. Except for mobility and hind limb clasping, all other symptoms showed significant improvement by VPA treatment. However mobility showed strong time and treatment interaction. (A) mobility (interaction: $F_{6,96}$ =5.657, $p < 0.0001$; VPA treatment: $F_{6,96}$ =2.840, $p = 0.113$); (B) gait (VPA treatment: $F_{1,96}$=8.544, $p = 0.01$); (C) hind limb clasping ($F_{1,96}$ = 2.45, $p = 0.137$); (D) tremor ($F_{1.96}$= 16.42, p =0.0009); (E) breathing ($F_{1,96}$ = 14.22, $p = 0.0017$); and (F) general health conditions ($F_{1,96}$ = 5.51, p = 0.0321). Data were analyzed using Two-way ANOVA with repeated measure. *, $p <$ 0.05; **, $p < 0.01$; ***, $p < 0.001$

Figure S2:

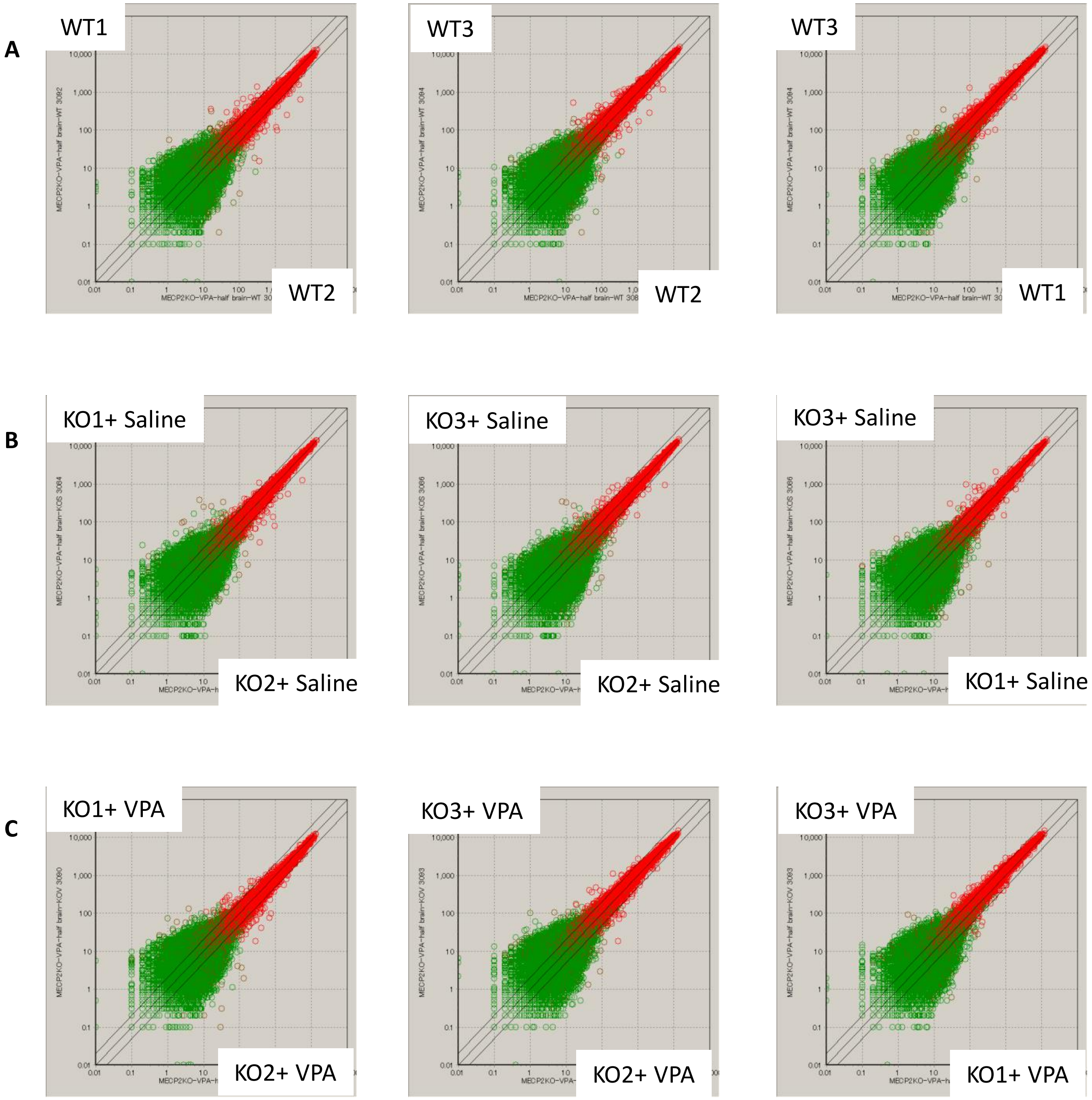

Figure S2. Scatter plots showing reproducibility in gene expression profiles among biological triplicates within each experimental condition.
(A) WT brains (B) KO + Saline (C) KO + VPA

Figure S3:

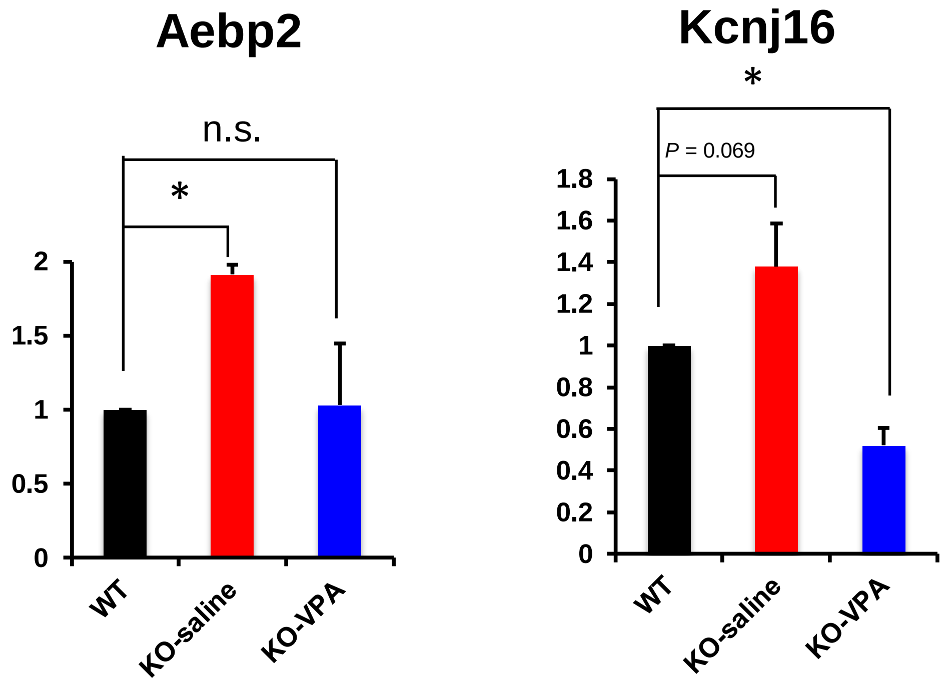

Figure S3. Quantitative PCR data showing restoration of Aebp2 and Kcnj16 genes in MeCP2 KO brains by VPA treatment
